# Reducing Stress Susceptibility of Broiler Chickens by Supplementing a Yeast Fermentation Product in the Feed or Drinking Water

**DOI:** 10.3390/ani8100173

**Published:** 2018-10-04

**Authors:** Jill R. Nelson, Don R. McIntyre, Hilary O. Pavlidis, Gregory S. Archer

**Affiliations:** 1 Department of Poultry Science, Texas A&M University, College Station, TX 77843, USA; jnelso15@gmail.com; 2 Diamond V, Cedar Rapids, IA 52404, USA; dmcintyre@diamondv.com (D.R.M.); hpavlidis@diamondv.com (H.O.P.)

**Keywords:** stress, broilers, Original XPC, AviCare, corticosterone, feed additive, yeast fermentation product

## Abstract

**Simple Summary:**

Poultry are susceptible to stress induced as a response to common management practices such as vaccination, feed withdrawal, and rearing on reused litter as well as severe stressors such as high environmental temperature. These stressors can result in economic costs to producers that are associated with poor growth and disease. Functional metabolites in the yeast fermentation product Original XPC^TM^ have previously shown positive effects on growth performance and immune function in a variety of livestock when incorporated into the diet. This study found that including either Original XPC^TM^ in the feed or its liquid counterpart, AviCare^TM^, in the drinking water during the entire rearing period can reduce stress and improve the welfare of broiler chickens under many stressful situations.

**Abstract:**

Reducing stress is an important goal in animal production. Previous research has demonstrated the ability of Original XPC^TM^ to reduce the stress response of broilers during heat stress. Three trials were conducted to evaluate the effects of adding Original XPC^TM^ to the feed or AviCare^TM^ to the water on stress susceptibility of broiler chickens. Treatments included: control nonstressed (CNS), control stressed (CS), stressed with Original XPC^TM^ (1.25 kg/metric ton feed, 0–42 days; XPC), and stressed with AviCare^TM^ (160 mL/100 L drinking water, 0–42 days; AVI). All stressed treatments received the following stressors: live coccidiosis vaccination (day 1), reared on reused litter (days 0–42), and heat stress with feed/water withdrawal (12 h on day 18). Plasma corticosterone and heterophil/lymphocyte (H/L) ratio were determined from 60 birds/T on day 19, and 24 birds/T on day 41. Physical asymmetry was determined using bilateral bone measurements from 60 birds/T on day 41. Birds provided XPC or AVI had lower corticosterone and H/L ratios than CS (*p* < 0.05) on day 19 and lower corticosterone, H/L ratios, and asymmetry scores than both CNS and CS on day 41 (*p* < 0.05) in all three trials. Supplementing XPC or AVI improved broiler welfare measured by reduced stress indicators after acute heat stress or normal rearing stress in all trials.

## 1. Introduction

Poultry are exposed to a multitude of environmental stressors in modern animal breeding, including vaccination, feed withdrawal, heat stress, high stocking density, and direct contact with feces in the litter. These factors can trigger the stress response, impair immunity, and introduce foreign pathogens into an otherwise healthy animal, thereby impacting normal growth and production. When a bird becomes stressed, the hypothalamic-pituitary-adrenal axis is activated, ultimately resulting in the secretion of corticosterone (CORT) into the bloodstream [1,2]. As the primary stress hormone in birds, CORT threatens bird health by suppressing immune responses [3], altering metabolism to increase readily available energy [1], slowing growth rate [4], and disrupting cecal microflora [5]. A variety of commercial growing practices can induce stress in poultry. Exposure to high temperatures and feed withdrawal is associated with greater susceptibility to pathogen colonization of the gut [5]. Heat stress can also result in poor feed intake, weight gain, and feed conversion ratio [4]. Moist litter also reduces welfare status through the development of footpad dermatitis [6] and increases the likelihood of birds coming into contact with microbes [7]. Overcrowding suppresses the immune system by reducing macrophage activity [8] and depresses growth rate [6], which is critical when broilers are expected to reach market weight in a typical 42-day rearing period. Finally, concomitant stressors can have an additive effect, impacting immune response or growth differently than if they were isolated incidents [9]. Thus, it is essential to mitigate stress during rearing in order to minimize morbidity and improve both production performance and animal welfare.

When added to the feed, the *Saccharomyces cerevisiae* fermentation product Original XPC^TM^ (XPC; Diamond V, Cedar Rapids, IA) has demonstrated effectiveness in improving growth performance and combating enteric infections in dairy calves [10], swine [11], and poultry—including laying hens [12], ducks [13], turkeys [14], and broilers [15,16]. The functional metabolites of the fermentation process, which are contained within XPC, can also be incorporated into the drinking water with the liquid product AviCare^TM^ (AVI, Diamond V, Cedar Rapids, IA, USA). Recent research has also shown that use of XPC can reduce physiological stress indicators in turkeys placed under short-term heat stress [17] and in broilers under long-term heat stress [18]. For example, Al-Mansour et al. showed that XPC significantly decreased the heterophil/lymphocyte (H/L) ratio in broilers [19]. In addition to the improvements in the immune and stress response, XPC has also been shown to have a positive impact on weight gain, feed conversion, and mortality in broilers [20,21]. This was even seen in broilers that were challenged with ingestion of used litter during a heat stress period [22].

The objective of this study was to determine whether XPC and AVI can reduce broiler chickens’ susceptibility to physiological stress when challenged with both an acute stress event and short-term stressors typical of a commercial 6-week broiler rearing program in the United States. It was hypothesized that supplementing broiler feed with XPC or adding AVI to the drinking water would decrease the stress susceptibility of broiler chickens subjected to acute stress and stress associated with management procedures during rearing.

## 2. Materials and Methods

### 2.1. Animal Husbandry

All procedures were carried out in accordance with the guidelines established by the Texas A&M Institutional Animal Care and Use Committee (AUP # 2016-0257) and the birds were managed according to the guidelines described in the Guide for the Care and Use of Agricultural Animals in Research and Teaching [23]. In total, three consecutive trials were conducted. Experimental design was similar in all three trials, and only the type of acute stress event varied among experiments. In each trial, 1200 healthy-looking day-of-hatch Cobb 500 male broilers were randomly assigned to one of four treatments, each with 12 replicates, for a total of 300 birds per treatment. Treatments consisted of: nonstressed control (CNS), stressed control (CS), stressed and treated with AVI at a rate of 160 mL/100 L drinking water continuously from days 0 to 42, and stressed and treated with XPC at a rate of 1.25 kg/metric ton (MT) of feed continuously from days 0 to 42. Pens measured 0.91 m × 1.83 m, allowing 0.067 m^2^ of floor space per bird from days 0 to 19 (25 birds per pen)—when five birds per pen were sacrificed for blood sampling—and 0.083 m^2^ per bird from days 20 to 42 (20 birds per pen). Pens were lined with 8–10 cm of pine shaving substrate, which was sourced from previous broiler grow-out trials. Building temperature was maintained at 31 °C for the first week of each trial, then at 29 °C for the second week, and subsequently reduced by 2.8 °C each week thereafter until an ambient temperature of approximately 23 °C was reached. Photoperiod consisted of 24 h of light for the first 3 days and 20 h of light followed by 4 h of darkness for the remainder of the trial.

One tube feeder and one drinker consisting of an 18.9-L bucket with four nipples on the bottom were hung in each pen and their heights adjusted as birds grew. Feed and water were provided ad libitum except during the 12-h fasting stress challenge on day 18 for CS, AVI, and XPC treatment groups. Birds received standard broiler diets which were mixed at the Texas A&M Poultry Research Center Feed Mill. Birds were fed a starter diet from days 0 to 19, a grower diet from days 20 to 28, and a finisher diet from days 29 to 42. AVI drinkers were emptied and refilled from a stock solution daily, and all others were refilled as needed. All birds, feeders, and drinkers for each pen were weighed at 8:00 p.m. on day 18 in each trial. Mortality rates were also recorded.

The birds in each pen were weighed on days 0 and 42 and body weight gain was calculated. Feed was weighed before its addition to the feeder in each pen and remaining feed was weighed on feed transition days so that total feed intake could be calculated. Feed conversion ratio was calculated by dividing the total feed intake per pen by the total body weight gain per pen, corrected for mortality. All birds were weighed on a per pen basis using a rolling scale (UFM-F120, UWE Scales, Cape Town, South Africa) on day 0 and individually using a hanging scale with shackles (RPBS-1, Rotem, Petach-Tikva, Israel) on day 42, and body weight gain was calculated.

### 2.2. Stress Challenges

Birds in the CS, AVI, and XPC pens were exposed to an acute stress challenge on day 18 in each trial. In Trial 1, birds were exposed to 12 h of heat stress produced by introducing a barrier to the pen in order to crowd birds and increase collective body heat, which was verified every 2 h by a maintained litter temperature of approximately 32 °C in these pens. The heat stress period began at 8:00 p.m. on day 18, after all birds, feeders, and drinkers were weighed for each pen, and ended at 8:00 a.m. on day 19. During this time period, the building lights remained on to mimic daylight conditions. At the end of the heat stress period, feeders and drinkers for CNS pens were weighed. Feeders and drinkers for all treatments were then refilled and returned to the respective pen. In Trial 2, birds were spray-vaccinated for Newcastle/Bronchitis (COMBOVAC-30®, Merck, Kenilworth, NJ, USA), but they retained free access to the entire pen. In Trial 3, birds in the CS, AVI, and XPC treatments received the Newcastle/Bronchitis vaccine and were exposed to the 12 h of heat stress as described in Trial 1. In addition, birds in CS, AVI, and XPC pens were exposed to stressors which may occur during the rearing period in industry conditions, including being reared on previously used litter and spray-vaccination for coccidiosis on day 1 (COCCIVAC®-B52, Merck, Kenilworth, NJ, USA). In all three trials, CNS birds maintained ad libitum access to feed and water during the 12-h acute stress period on day 18 and were neither exposed to crowding-heat stress conditions nor did they receive vaccination for coccidiosis on day 1 or Newcastle/Bronchitis on day 18.

### 2.3. Stress Measures

In each trial, blood samples were collected via exsanguination following decapitation from five birds per pen (*n* = 60 per treatment) on day 19 following the 12-h acute stress challenge. Blood samples were also collected via wing vein venipuncture from two birds per pen (*n* = 24 per treatment) on day 41. One to two milliliters of blood from each bird were transferred to a spray-coated lithium heparin and polymer separation gel vacutainer (368056, BD Medical, Franklin Lakes, NJ, USA). Vacutainers were stored in an ice bath while remaining blood samples were collected. A drop of blood from each sample was used to make a blood smear slide. Vacutainers were spun down at 4000 RPM for 15 min (Centrifuge 5804, Eppendorf, Hamburg, Germany). The plasma layer was then poured off into a labeled 2-mL microcentrifuge tube and stored at -20 °C; samples were thawed overnight at 4 °C prior to assay. Plasma corticosterone concentration was obtained using a 96-well commercial ELISA kit (ADI-901-097, Enzo Life Sciences, Inc., Farmingdale, NY, USA). Absorbance was measured at 450 nm using a microplate absorbance reader (Tecan Sunrise, Tecan Trading AG, Männedorf, Switzerland) and analyzed using the Magellan Tracker software program. Dry blood smear slides were stained with a neat stain hematology stain kit (Cat. #25034, Poly Sciences, Inc., Warrington, PA, USA) and used to determine H/L ratio at 40× magnification using an oil immersion lens under microscopy (89404-886, VWR International, Radnor, PA, USA).

On day 41, bilateral bone trait measurements were collected from five birds per pen (*n* = 60 per treatment). Middle toe length (MTL), metatarsal length (ML), and metatarsal width (MW) in millimeters were obtained on the left (L) and right (R) legs using Craftsman IP54 digital calipers (Sears Holdings, Hoffman Estates, IL). A composite asymmetry score (ASYM) for each bird was calculated using the following formula:

(|L-R|_MTL_ + |L-R|_ML_ + |L-R|_MW_) ÷ 3 (1)

### 2.4. Statistical Analysis

Data for stress measures, mortality, body weight, and feed conversion ratio were analyzed using the General Linear Model in Minitab 17.1.0 (Minitab, Inc, State College, PA, USA). Data were compared using one-way analysis of variance (ANOVA), with treatment as the only effect. Mean separation was performed using the post hoc test for least significant differences (LSD). A significant difference was defined as *p* < 0.05. Data were not transformed for analysis.

## 3. Results

### 3.1. Plasma Corticosterone

Data for CORT in all three trials are presented in Table 1. Treatment affected plasma CORT on day 19 following acute stress in all three trials (Trial 1: F_3,236_ = 22.87, *p* < 0.001; Trial 2: F_3,236_ = 3.03, *p* < 0.03; Trial 3: F_3,236_ = 4.85, *p* < 0.003), with CS birds displaying significantly higher plasma CORT values compared to the other groups (Trial 1: *p* < 0.001; Trial 2: *p* < 0.05; Trial 3: *p* < 0.03). Treatment also affected plasma CORT levels on day 41 in all three trials (Trial 1: F_3,92_ = 3.66, *p* = 0.02; Trial 2: F_3,92_ = 4.77, *p* = 0.004; Trial 3: F_3,92_ = 3.47, *p* = 0.02). On day 41, both AVI (Trial 1: *p* < 0.04; Trial 2: *p* < 0.05; Trial 3: *p* < 0.03) and XPC (Trial 1: *p* < 0.02; Trial 2: *p* < 0.05; Trial 3: *p* < 0.03) had lower plasma CORT than CNS and CS groups in all three trials. However, significant differences between AVI and XPC in CORT levels on day 41 were not observed (*p* > 0.05).

### 3.2. H/L Ratio

Data for H/L ratios for all trials are presented in Table 2. Treatment affected H/L ratio on day 19 following acute stress in all three trials (Trial 1: F_3,236_ = 5.85, *p* = 0.001; Trial 2: F_3,236_ = 3.46, *p* = 0.02; Trial 3: F_3,236_ = 3.46, *p* = 0.02), with CS birds displaying significantly higher plasma CORT values compared to the other groups (Trial 1: *p* < 0.02; Trial 2: *p* < 0.04; Trial 3: *p* < 0.04). Treatment affected H/L ratio at day 41 in all three trials (Trial 1: F_3,92_ = 4.10, *p* = 0.009; Trial 2: F_3,92_ = 2.96, *p* = 0.04; Trial 3: F_3,92_ = 3.21, *p* = 0.02). On day 41, both AVI and XPC (Trial 1: *p* < 0.04; Trial 2: *p* < 0.05; Trial 3: *p* < 0.03) had lower H/L ratios than the CNS and CS groups in all three trials. However, significant differences between AVI and XPC birds in H/L ratio on day 41 were not observed (*p* > 0.05).

### 3.3. Physical Asymmetry

Composite asymmetry scores for all trials are presented in Table 3. Treatment affected composite asymmetry score in all three trials (Trial 1: F_3,236_ = 3.78, *p* = 0.01; Trial 2: F_3,236_ = 3.32, *p* = 0.02; Trial 3: F_3,236_ = 5.51, *p* = 0.001). Composite asymmetry scores were lower in AVI (Trial 1: *p* < 0.03; Trial 2: *p* < 0.05; Trial 3: *p* < 0.003) and XPC (Trial 1: *p* < 0.04; Trial 2: *p* < 0.03; Trial 3: *p* < 0.01) compared to CNS and CS in all three experiments. There was no difference in composite asymmetry score between CNS and CS (*p* > 0.05) in any trial.

### 3.4. Mortality

There was no effect of treatment on mortality in all three experiments (*p* > 0.05). Average cumulative mortality was 6.7%, 2.1%, and 4.9% in Trials 1, 2, and 3 respectively.

### 3.5. Body Weight

Body weight data for all experiments are presented in Table 4. Body weight on day 42 did not significantly differ (F_3,44_ = 1.21, *p* = 0.32) between treatments in Trial 1, although there was a trend (*p* = 0.08) toward heavier birds in the XPC group when compared to the CS group. In Trial 2, treatment had an effect on day-42 body weight (F_3,44_ = 3.22, *p* = 0.03). Both AVI (*p* = 0.01) and XPC (*p* = 0.01) had heavier day-42 body weights than CNS, while CS remained intermediate between them. In Trial 3, treatment had an effect on day-42 body weight (F_3,44_ = 15.76, *p* < 0.001). However, the only effect of treatment on day-42 body weight was seen in CS birds, which weighed less than all other treatments (*p* < 0.001).

### 3.6. Feed Conversion Ratio (FCR)

Feed conversion ratio data for all experiments are presented in Table 4. Feed conversion did not significantly differ among treatments (F_3,44_ = 1.35, *p* = 0.27) in Trial 1, although there was a slight trend (*p* = 0.09) toward lower FCR in XPC compared to CS. In Trial 2, treatment had an effect on FCR (F_3,44_ = 2.89, *p* = 0.046). XPC had lower (*p* < 0.02) feed conversion than all other treatments. In Trial 3, treatment had an effect on FCR (F_3,44_ = 4.07, *p* = 0.01). AVI had lower feed conversion compared to CNS (*p* = 0.003) and CS (*p* < 0.007), while XPC did not significantly differ (*p* > 0.05) from the other treatments.

Plasma corticosterone and H/L ratio are generally used for stress assessment in birds [18,19]. In this study, birds in the CS group had significantly elevated CORT and H/L ratio compared to CNS birds as a result of the imposed acute stress challenge on day 18 in all three trials. In addition, XPC and AVI birds had lower H/L ratios and CORT levels than CS birds following the imposed acute stress on day 18. These results are in line with Price et al., who showed that XPC supplementation reduced CORT levels, H/L ratios, and ASYM scores in broilers during cyclic heat stress [18]. Composite asymmetry score can be used to measure an animal’s ability to cope with stress and direct energy toward normal growth over the long-term period [24]. XPC and AVI birds had lower composite asymmetry scores after 41 days of growth than CNS and CS birds in all three trials. Smaller relative ASYM scores indicate equal growth of bilateral traits over time and, therefore, an enhanced ability to shuttle energy toward growth in spite of exposure to a variety of environmental stressors. XPC and AVI birds also had lower CORT levels and H/L ratios than CNS and CS birds on day 41, which is in agreement with a previous study showing that XPC reduced H/L ratios in broilers during normal rearing stress [19]. The numerical differences between CORT and H/L measures between trials can be attributed to differing external conditions such as weather, as even though the barns were tunnel ventilated, temperature and humidity within them can still be affected by time of year weather conditions. Birds in the AVI and XPC groups were less susceptible to physiological stress than untreated birds under the same conditions over the course of the grow-out period, and as a result, presented more symmetrical growth of bilateral bone traits at 42 days of age. A proposed explanation for this is a possible immunomodulatory effect of yeast fermentation metabolites on the intestine [14]. Future research could further explore the effects of ingestion of yeast fermentation metabolites on intestinal health and immune response and its relation to stress susceptibility in poultry.

Birds supplemented with XPC had improved FCR compared with all other treatments in Trial 2, and in Trial 3, those supplemented with AVI had lower FCR compared with all other treatments, whereas XPC birds were statistically intermediate between the control treatments and AVI. Previous experiments have demonstrated better feed conversion in male turkeys raised on used litter [14] and improved FCR and weight gain in broilers [25] when XPC is administered continuously in the diet. Birds that are less susceptible to environmental stress may experience improved nutrient absorption and may be more inclined to shuttle energy toward tissue accretion rather than tissue turnover [19]. It has also been shown that the product of *S. cerevisiae* fermentation can help control inflammation and improve antioxidant status, both of which are results of acute heat stress [12]. Both AVI and XPC birds had consistently lower CORT and H/L ratios after exposure to acute heat stress in this study, which does not explain why differences in FCR among treatments were not consistent across all three trials. Although there is evidence that XPC improves feed efficiency in broilers exposed to acute and rearing stressors, a larger-scale investigation may clarify any trends in feed conversion when birds are supplemented with XPC compared to AVI.

All stressed birds in this study received a live coccidiosis vaccine at the beginning of each trial. Previous studies have shown that XPC improves growth in broilers challenged with coccidial infection [20] or administered a live coccidiosis vaccine [26]. This agrees with some results regarding measures of growth in this study: for example, XPC and AVI exhibited similar day-42 body weights to CNS birds in Trial 3, and XPC birds tended to have a greater day-42 body weight than all other treatments in Trial 1. However, when birds were challenged with an additional Newcastle/Bronchitis vaccination in Trial 2 in this study, there was no effect of treatment on day-42 body weight. Although XPC and AVI birds showed improved growth of bilateral traits compared to CS and CNS birds in Trial 2, this improvement in metabolic homeostasis was not reflected in a significantly greater overall gain.

## 5. Conclusions

Both XPC and AVI reduced stress equally across all three experiments, as evidenced by H/L ratios, CORT levels, and ASYM score. Differences in the effects of these products on feed conversion and day-42 body weight could be elucidated by future studies, including exploration of the relationship between growth performance and inclusion level or mode of administration. The effects of XPC have been broadly demonstrated in other livestock species. This study provides information on its effects on additional measures of stress susceptibility in broiler chickens and explores the effects of AVI when administered continuously in the drinking water during the entire rearing period. To conclude, inclusion of either Original XPC in the feed or AviCare in the drinking water during the entire rearing period reduces broiler susceptibility to acute stress events and stress induced by management practices during rearing.

## Figures and Tables

**Table 1:** Plasma corticosterone (pg/mL) after an acute stress challenge (day 19) and after 41 days of growth.

Treatment	Trial 1		Trial 2		Trial 3	
	Day 19 ^1^	Day 41 ^2^	Day 19 ^1^	Day 41 ^2^	Day 19 ^1^	Day 41 ^2^
CNS	194.3 ^a^	233.7 ^a^	1042.9 ^a^	5449.9 ^a^	580.5 ^a^	1684.7 ^a^
CS	690.9 ^b^	252.0 ^a^	1726.5 ^b^	5385.8 ^a^	973.0 ^b^	1625.0 ^a^
AVI	313.0 ^a^	75.0 ^b^	1030.7 ^a^	2482.4 ^b^	559.3 ^a^	468.3 ^b^
XPC	279.6 ^a^	56.3 ^b^	1081.7 ^a^	2424.1 ^b^	312.6 ^a^	518.1 ^b^
Pooled SEM^3^	26	28	117	354	63.4	187
Treatment Main Effect *p*-Value	< 0.001	0.02	0.03	0.004	0.003	0.02

^1^*n* = 60 birds per treatment,^2^ *n* = 24 birds per treatment; ^a,b^ Values within a column with different superscripts differ significantly at *p* < 0.05. CNS: nonstressed control; CS: stressed control; ^3^Standard error of the mean (SEM)

**Table 2:** Heterophil/lymphocyte ratios after an acute stress challenge (day 19) and after 41 days of growth.

Treatment	Trial 1		Trial 2		Trial 3	
	Day 19^1^	Day 41^2^	Day 19^1^	Day 41^2^	Day 19^1^	Day 41^2^
CNS	0.10^a^	0.25^a^	0.09^a^	0.21^a^	0.10^a^	0.29^a^
CS	0.21^b^	0.25^a^	0.14^b^	0.20^a^	0.16^b^	0.28^a^
AVI	0.15^a^	0.16^b^	0.10^a^	0.12^b^	0.12^a^	0.22^b^
XPC	0.13^a^	0.16^b^	0.09^a^	0.11^b^	0.11^a^	0.21^b^
Pooled SEM^3^	0.01	0.01	0.01	0.02	0.01	0.01
Treatment Main Effect *p*-Value	0.001	0.009	0.02	0.04	0.02	0.02

^1^*n* = 60 birds per treatment,^2^ *n* = 24 birds per treatment; ^a, b^ Values within a column with different superscripts differ significantly at *p* < 0.05. CNS: nonstressed control; CS: stressed control; ^3^Standard error of the mean (SEM).

**Table 3:** Composite asymmetry scores (mm) after 41 days of growth ^1^.

Treatment	Trial 1	Trial 2	Trial 3
CNS	2.13 ^a^	2.48 ^a^	2.94 ^a^
CS	2.06 ^a^	2.41 ^a^	2.90 ^a^
AVI	1.68 ^b^	2.01 ^b^	2.23 ^b^
XPC	1.70 ^b^	1.98 ^b^	2.33 ^b^
Pooled SEM^3^	0.06	0.07	0.08
Treatment Main Effect *p-*Value	0.01	0.02	0.001

^1^*n* = 60 birds per treatment; ^a, b^ Values within a column with different superscripts differ significantly at *p* < 0.05. CNS: nonstressed control; CS: stressed control; ^3^Standard error of the mean (SEM).

**Table 4:** Average individual body weight (kg) from all birds at 42 days of age and cumulative feed conversion ratio from days 0 to 42.

Treatment	Day 42 Body Weight (kg)			Feed Conversion Ratio ^1^		
	Trial 1	Trial 2	Trial 3	Trial 1	Trial 2	Trial 3
CNS	2.41	2.65 ^a^	2.85 ^a^	1.757	1.533 ^a^	1.693 ^a^
CS	2.36	2.70 ^ab^	2.61 ^b^	1.745	1.528 ^a^	1.680 ^a^
AVI	2.44	2.76 ^b^	2.83 ^a^	1.702	1.528 ^a^	1.547 ^b^
XPC	2.47	2.76 ^b^	2.81 ^a^	1.679	1.449 ^b^	1.617 ^ab^
Pooled SEM^3^	0.02	0.02	0.02	0.02	0.01	0.02
Treatment Main Effect *p*-Value	0.32	0.03	<0.001	0.27	0.046	0.01

^1^ Cumulative feed conversion ratio adjusted for mortality; ^a, b^ Values within a column with different superscripts differ significantly at *p* < 0.05. CNS: nonstressed control; CS: stressed control; ^3^Standard error of the mean (SEM).4. Discussion
